# Legislative advocacy and public policy work for academic and research library workers: perspectives and strategies

**DOI:** 10.29173/jchla29908

**Published:** 2026-04-01

**Authors:** Amy Rutherford

**Affiliations:** Health Sciences Librarian Queen’s University, Kingston, ON, Canada

Pun R, Durney SM, Anantachai T, editors. **Legislative advocacy and public policy work for academic and research library workers: perspectives and strategies**. 1st ed. Chicago, IL: Association of College & Research Libraries; 2025. Softcover: 296 p. ISBN: 979-8-89255-627-9. Price: USD$90.00. Available from: https://alastore.ala.org/legislative-advocacy-and-public-policy-work-academic-and-research-library-workers-perspectives-and.

*Legislative advocacy and public policy work for academic and research library workers: perspectives and strategies* [1], brings together a diverse group of advocacy experts for a broad, in-depth discussion of library advocacy and the ways that academic and research librarians are participating in, promoting, and raising awareness for library advocacy in the academic and governmental sectors. Edited by Pun, Durney, and Anantachai, this book primarily focuses on American perspectives of legislative advocacy, while also featuring four chapters by Canadian librarians that address Canadian advocacy issues.

The editors are academic librarians working in higher education. Raymond Pun is an Academic and Research Librarian at Alder Graduate School of Education in California [2]. Sonya M. Durney is a Scholarly Communications Librarian at University of New England [2], and Tarida Anantachai, a librarian by profession, is currently the Director of Talent Management at North Carolina State University Libraries [2], also named a “Change Agent” by the *Library journal* in 2024 [3].

A brief search in Queen’s University Library catalogue for “advocacy and libraries / librarians” showed there are numerous titles on advocacy for public and school librarians, but few covering advocacy in the academic and research sectors. This book fills that gap as each chapter looks at advocacy issues affecting academic librarians and library workers at universities and colleges.

The chapter authors provide their expertise in library advocacy through a multitude of case studies and practical examples of advocacy work. This book includes an introduction, and 22 chapters which are divided into two sections. Section one focuses on public policy issues affecting academic and research libraries. Section two explores perspectives, practical suggestions, and tips shared by academic librarians engaged in advocacy work. Although the book is American focused, many of the strategies and real-life examples brought forward could be used and adapted to a Canadian context.

The Canadian chapters address topics such as Canadian copyright law, broadband access for rural and remote communities, the history and significance of GovInfo Days, and a discussion of relationship building between politicians and librarians. Additional advocacy issues and the potential approaches addressed include open education resources (OERs), digital equity, accessibility, American copyright law, government lobbying at state and federal levels, intellectual freedom, and diversity, equity, and inclusion (DEI) programs.

The writing style, tone, and reading level of the chapters are consistent, accessible, and easy to understand. Each chapter includes notes and references at the end, so one does not have to refer to endnotes while reading. Adding a glossary of acronyms would have been a helpful addition, as some chapters that delve into the specifics of state government legislation include an overwhelming number of acronyms that sometimes impede the understanding of the content.

An innovative aspect of this book is the multiple chapters presented in interview format. The interviews are interspersed throughout, adding an impactful energy to the text. The editors speak with librarians who are involved in advocacy work, allowing their voices to convey their passion, expertise, and practical insights drawn directly from experience. Notable interviewees include Frans Albarillo, advocating for digital privacy and privacy literacy instruction for students (chapter seven), Arnetta Girardeau, emphasizing her positionality as a person of colour, which drives her civil rights advocacy through her work as both a lawyer and copyright librarian (chapter 18), and Ken Haycock asserting the power of relationship building with politicians to be successful in library advocacy (chapter 13). Haycock was Director of the iSchool at University of British Columbia and at San José State University, providing both Canadian and American perspectives. Haycock’s statement that “the relationship is the message” [4] is a key theme, which numerous chapter authors echo throughout the book. This interview would have made a strong introduction, to inspire readers to engage with the rest of the book.

Numerous chapters focus extensively on state government advocacy with in-depth details of government processes. For example, Horne, in chapter nine, gives a detailed description of state legislative websites and the various strategies librarians can utilize to obtain current information on bills and how they move through the legislature. The chapters focusing on U.S. government processes specifically are less relevant to a Canadian audience, but the overall advocacy recommendations can still be useful.

Major themes that emerge in the book include lobbying within the confines of the academic setting, relationship building with politicians to affect library budgets and funding opportunities, and adding a variety of advocacy and political training to Library and Information Studies (LIS) programs’ curricula to improve advocacy and lobbying skills. In chapter five, Malenfant, Blandino, and Craig [1] discuss the Digital Equity Act (DEA) which is supposed to provide “$2.75 billion over five years to promote digital equity, literacy and inclusion initiatives…” [5] across the U.S. They note that the funding for the DEA was included in one of President Trump’s executive orders in January 2025 [6]. Another example is Whitledge’s (chapter 12) discussion of humanities funding for archives through the Institute of Museum and Library Services (IMLS). Librarians must remain vigilant and committed to advocacy because of the reliance on government funding, highlighting the significance of librarian lobbyists strengthening political relationships to increase government funding and library budgets. Returning to Haycock’s point, building the relationships with politicians is essential for successful library advocacy.

In chapter 15, Walz presents in-depth analysis of her experiences as a “librarian lobbyist” [7] working to change the language of an open educational resources (OER) Virginia State bill. Her chapter is a strong example of the aforementioned themes. She reflects honestly about her mistakes so readers can learn from her experience. She made a significant error by not consulting the university relations and leadership at her institution. She acknowledges that the lobbying messaging must come from higher levels of the university. Walz also notes that political skills development should be incorporated into LIS education so that librarians can be more effective political lobbyists in future [8].

Joseph and McNally (chapter 16) also make a case that Canadian LIS education should incorporate the roles that libraries and library workers play in providing broadband access for rural and remote areas of Canada. They note that Canada has fallen behind, and improved educational programming is one factor in addressing this issue.

Overall, *Legislative advocacy and public policy work for academic and research library workers: perspectives and strategies* present a hopeful and positive view of library advocacy work in academic libraries across the United States and Canada. This book would be recommended as an inspirational and educational addition for any academic library collection. It covers a diverse range of advocacy topics with practical examples and advice from experienced librarian advocates. As the current U.S. government administration is focusing attacks on programs highlighted in this book such as DEI programs, intellectual freedom, digital equity, and the humanities, library advocacy will be essential work for present and future librarians.
